# Rho Family GTPases and Rho GEFs in Glucose Homeostasis

**DOI:** 10.3390/cells10040915

**Published:** 2021-04-16

**Authors:** Polly A. Machin, Elpida Tsonou, David C. Hornigold, Heidi C. E. Welch

**Affiliations:** 1 Signalling Programme, The Babraham Institute, Babraham Research Campus, Cambridge CB22 3AT, UK; polly.machin@babraham.ac.uk (P.A.M.); elpida.tsonou1@astrazeneca.com (E.T.); 2 Bioscience Metabolism, Research and Early Development, Cardiovascular, Renal and Metabolism (CVRM), BioPharmaceuticals R&D, AstraZeneca, Cambridge CB22 3AT, UK; David.Hornigold@astrazeneca.com

**Keywords:** Rho GTPase, small G protein, Rho GEF, guanine nucleotide exchange factor, glucose homeostasis, metabolic syndrome, type 2 diabetes, GLUT4 glucose transporter, insulin-stimulated glucose uptake, glucose-stimulated insulin secretion

## Abstract

Dysregulation of glucose homeostasis leading to metabolic syndrome and type 2 diabetes is the cause of an increasing world health crisis. New intriguing roles have emerged for Rho family GTPases and their Rho guanine nucleotide exchange factor (GEF) activators in the regulation of glucose homeostasis. This review summates the current knowledge, focusing in particular on the roles of Rho GEFs in the processes of glucose-stimulated insulin secretion by pancreatic β cells and insulin-stimulated glucose uptake into skeletal muscle and adipose tissues. We discuss the ten Rho GEFs that are known so far to regulate glucose homeostasis, nine of which are in mammals, and one is in yeast. Among the mammalian Rho GEFs, P-Rex1, Vav2, Vav3, Tiam1, Kalirin and Plekhg4 were shown to mediate the insulin-stimulated translocation of the glucose transporter GLUT4 to the plasma membrane and/or insulin-stimulated glucose uptake in skeletal muscle or adipose tissue. The Rho GEFs P-Rex1, Vav2, Tiam1 and β-PIX were found to control the glucose-stimulated release of insulin by pancreatic β cells. In vivo studies demonstrated the involvement of the Rho GEFs P-Rex2, Vav2, Vav3 and PDZ-RhoGEF in glucose tolerance and/or insulin sensitivity, with deletion of these GEFs either contributing to the development of metabolic syndrome or protecting from it. This research is in its infancy. Considering that over 80 Rho GEFs exist, it is likely that future research will identify more roles for Rho GEFs in glucose homeostasis.

## 1. Rho Family GTPases and Rho GEFs

The Rho family of Small Guanosine Triphosphatases (GTPases) are part of the Ras GTPase superfamily and were discovered through their homology to the Ras GTPases [1,2,3]. The Rho family consists of twenty members in mammals, and is best known for controlling the actin cytoskeleton and cell morphology [4]. The best-characterized members are RhoA whose activation leads to stress fiber formation (F-actin bundles), Cdc42 which induces filopodia formation (F-actin bundles ‘fingers’) and Rac1 which leads to lamellipodia formation (F-actin sheets) [5]. The Rho GTPases are also known to influence cell polarity, membrane trafficking, and microtubule formation. They also affect processes that may be unrelated to cytoskeletal dynamics, such as transcription factor activity [6], cell cycle control [7], and reactive oxygen species production [8]. It is not surprising that dysregulation of this protein family can lead to serious health problems and that it is an essential candidate for further research.

Diverse new roles have emerged recently for the Rho GTPase family in whole-body metabolic processes, including glucose homeostasis and insulin signaling. Incidences of metabolic diseases have been rising for several decades in the Western world, including metabolic syndrome, which encompasses a plethora of metabolic abnormalities including obesity, insulin resistance, hypertension and dyslipidemia. It is thought that 25% of the UK population suffer from metabolic syndrome, with type 2 diabetes mellitus and cardiovascular disease being among the leading causes of death in these patients. Studying the Rho GTPases in this context may further our understanding of metabolic disorders and could lead to new therapeutics. This review will focus on the Rho GTPases, and in particular their activators, guanine nucleotide exchange factors (GEFs), emphasizing insights into the known and emerging roles that these proteins play in glucose metabolism in vivo, as well discussing insights from in vitro studies of primary cells or cell lines derived from the pancreas, skeletal muscle and adipose tissue.

### 1.1. Regulation of Rho GTPases

**Figure 1:**
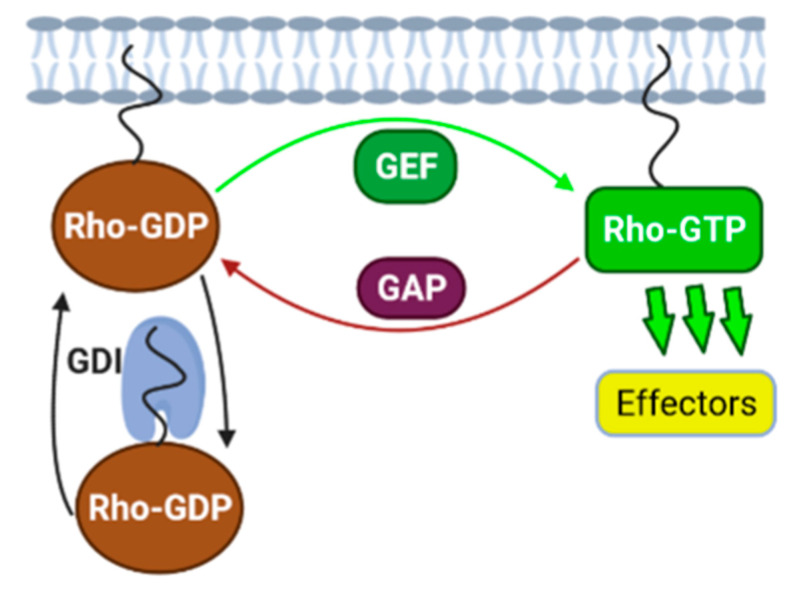
Regulation of Small GTPase activity. The activity of small GTPases, including the Rho family, is regulated by guanine nucleotide exchange factors (GEFs), GTPase-activating proteins (GAPs), and guanine nucleotide dissociation inhibitors (GDIs). GEFs activate Rho GTPases by promoting GTP loading. The active, GTP-bound form of the Rho GTPase adopts a conformation that allows it to interact with effector proteins. GAPs inactivate Rho GTPases by stimulating GTP hydrolysis. GDIs prevent the activation of Rho GTPases by promoting cytosolic localization. Figure made with BioRender.

Small GTPases, also known as small guanine nucleotide-binding proteins (G proteins), are monomeric molecular switches involved in signal transduction. They respond to upstream signals by conformational change and transduce the signal by binding to downstream effectors. Small GTPases are inactive in their guanosine diphosphate (GDP)-bound form and active in their guanosine triphosphate (GTP)-bound form (Figure 1). guanine nucleotide exchange factors (GEFs) hold the GTPase in a conformation that has a low affinity for nucleotides [9]. The GDP is released and GTP is then bound to the GTPase due to the naturally high intracellular concentration of GTP. In the GTP-bound, active state, the GTPase adopts a conformation that allows it to interact with its effector proteins, thus transmitting signals downstream [10]. GTPase-activating proteins (GAPs) turn off the signal through increasing the intrinsic GTPase activity of the Small GTPase, leading to GTP hydrolysis to GDP. Some small GTPases, including the Rho family, are additionally regulated by guanine nucleotide dissociation inhibitors (GDIs), which sequester the inactive GTPase in the cytosol and prevent it from binding to the plasma membrane [11]. There are two flexible regions, switch I and switch II, which are critical for the biological functions of small GTPases, as they confer the guanine nucleotide-sensitive conformational change [12]. The mechanism for the Rho switch is simple, but it is carefully regulated by over 80 known activating GEFs and 70 deactivating GAPs. This leads to a highly complex biological system where spatiotemporal regulation of Rho family GTPases can lead to the coordinated activation of several diverse signaling pathways.

Rho GTPase activity is regulated by mechanisms aside from GEF-GTP/GAP-GDP cycling [9]. Small GTPases, including the Rho family, are generally post-translationally lipid-modified by prenylation to tether the active protein to membranes. The C-terminal CAAX motif is recognized by transferases for the addition of farnesyl (15-carbon chain) or geranylgeranyl (20-carbon chain) groups at the cysteine residue [1,13,14]. Other lipid moiety modifications to Rho proteins include palmitoylation, and some Rho family GTPases also contain a C-terminal polybasic Lys/Arg region that targets the positively charged interface region to the membrane. The targeting of active Rho family GTPases to membranes leads to spatiotemporal regulation of Rho GTPase activity. The exceptions are RhoBTB1 and RhoBTB2, which are tumor suppressers that are not modified by lipids [15]. Rho-GTPases are also regulated at the level of gene expression, e.g., by microRNAs, or by a host of post-translational modifications, including ubiquitination, sumoylation and phosphorylation [16,17,18,19,20]. Phosphorylation often occurs close to lipid modifications, leading to a change in the localization of the GTPase, whereas ubiquitination leads to targeting degradation of the GTPase. Sumoylation is not thought to be essential, but helps to maintain the GTPase in an active state [9,21]. 

### 1.2. Rho GTPases and Their Downstream Effects 

The earliest described function of Rho GTPases was their regulation of the actin cytoskeleton. The activation of various membrane receptors by growth factors, including epidermal growth factor, insulin and platelet-derived growth factor, were shown to induce the formation of lamellipodia and membrane ruffling in Swiss 3T3 fibroblast cells, which could be blocked using dominant-negative N17-Rac1 [22]. A separate study in the same cell line showed that treatment with bradykinin-induced filopodia formation, and the effects were specifically inhibited by dominant-negative N17-Cdc42 [23]. Finally, a role for RhoA was discovered in focal adhesion and contractile actomyosin-rich stress fiber formation, in Swiss 3T3 fibroblasts upon stimulation with lysophosphatidic acid [22,24]. This was blocked by the addition of C3 transferase, a toxin from *Clostridium botulinum* that inhibits RhoA through ADP-ribosylation. Since these early studies, Rho family proteins have been shown to be the most important regulators of cytoskeletal dynamics, in processes as varied as cell polarity, chemokinesis and chemotaxis, phagocytosis and axon guidance. Rho family proteins are not limited to their effect on the cytoskeleton. Through multiple downstream effectors in a number of different signal transduction pathways, they also regulate NADPH oxidase activity in phagocytes [25,26], as well as cycle progression [7], cell proliferation [27,28,29,30,31] and gene transcription pathways [32,33,34], highlighting an important area for cancer research [35]. 

Rac, Cdc42 and RhoA are the best-characterized Rho family GTPases. There are four Rac-like proteins: Rac1 and RhoG are ubiquitously expressed, whereas Rac2 is restricted to the hematopoietic system and Rac3 to the nervous system [36]. Major Rac effectors include the WASP-family scaffold protein WAVE [37], which links upstream signals to the activation of the Arp2/3 complex, leading to the polymerization of branched actin filaments [38]. Another major Rac effector is the serine/threonine protein kinase PAK [39,40,41], which leads to actin polymerization, actomyosin contraction, microtubule stability [42] and activation of the ERK signaling pathway [43,44]. In humans, there are six PAKs, organized into group I (PAKs 1–3) and group II (PAKs 4–6) [45]. PAKs activate LIM domain kinases (LIMKs), which catalyze the inactivating phosphorylation of cofilin, an actin severing protein that leads to actin depolymerization [46]. Conversely, PAK1 can also act as an upstream activator of Rac1, by interacting with the Rac-GEF PAK-interacting exchange factor (PIX) [47]. PAK1 and PAK2 are the only group I isoforms expressed in skeletal muscle [48,49], and they have both been implicated in insulin-stimulated skeletal muscle glucose uptake. PAK1 whole-body deficiency in mice leads to impaired glucose tolerance and insulin sensitivity in vivo [50]. In addition, several studies have linked both PAK1 and PAK2 to the regulation of glucose homeostasis [51,52,53]. However, a recent study by the Sylow lab challenges this, suggesting that insulin-stimulated glucose uptake relies partly on PAK2, but not PAK1 [54]. Glucose tolerance in vivo and the insulin-stimulated uptake of 2-deoxy-D-glucose into extensor digitorum longus muscle ex vivo were mildly impaired in mice lacking PAK2, but not PAK1, using muscle-specific deletion [54]. In addition to WAVE and PAKs, Rac1 also activates the cofilin phosphatase slingshot1 (Ssh1), leading to cofilin inactivation [55]. Other Rac effectors include MLK [56], which leads to activation of the JNK pathway and AP1 transcription factor-dependent gene expression, and p67^phox^, a member of the NADPH oxidase complex and integral part of ROS production [36,57,58].

Rac and Cdc42 have overlapping sets of downstream effectors due to the minimal consensus Cdc42/Rac-binding motif known as the CRIB domain, to which both GTPases bind in their active, GTP-bound conformation [56]. There are two isoforms of Cdc42 produced by alternative splicing. Isoform 1 or Cdc42a is ubiquitously expressed, whereas isoform 2 or Cdc42b is restricted to the brain. Cdc42 is best known for inducing actin polymerization in filopodia through binding to WASP [59,60], or the insulin receptor substrate p53 (IRSp53) kinase to induce filamentous actin through Arp2/3 complex [61,62,63]. Actin polymerization is also induced through activation of mammalian diaphanous2 (mDia2), which is important for maintaining a stable pool of F-actin at the lamella [64]. 

RhoA is ubiquitously expressed, and activates Rho-associated protein kinase (ROCK) to mediate stress-fiber formation through phosphorylation of MLC and inactivation of MLC phosphatase, as well as through activation of LIMK, which phosphorylates cofilin to stabilize actin filaments [65,66,67]. The diaphanous-related formins (DRFs) are other critical downstream effectors of RhoA, whose activation leads to actin nucleation acceleration due to removal of barbed-end capping proteins [68]. Crosstalk between Rho GTPases is common. An early example in fibroblasts suggested that there is a linear hierarchical relationship where Cdc42 activates Rac and Rac activates Rho [69]. The relationship between these Rho family GTPases is not as linear as first thought, and more complex, even reciprocal interactions between Rac and RhoA are frequently observed. For example, Rac activation leads to RhoA inhibition during neurite outgrowth [70].

### 1.3. Rho GEFs 

**Figure 2:**
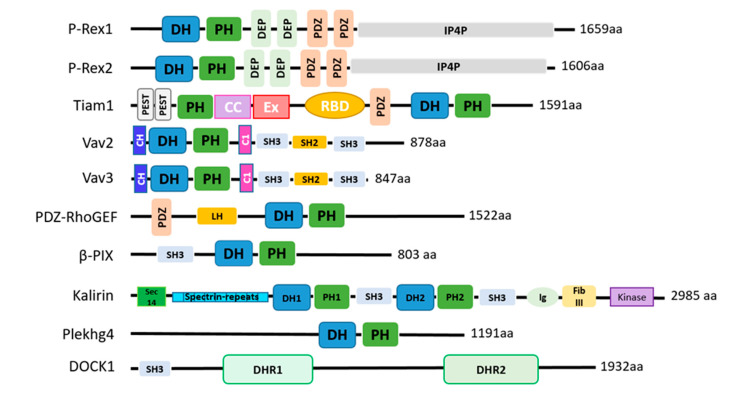
Domain structure of Rho GEFs involved in glucose homeostasis. The Rho GEFs are classified into two families, 70 Dbl-type and 11 DOCK-type proteins in mammals. This figure depicts the domain structures of the nine mammalian Dbl-type GEFs and of yeast Dck1, a homologue of mammalian DOCK1, which have been implicated to date in the regulation of glucose homeostasis. Dbl-type Rho GEFs are characterized by a catalytic Dbl homology (DH) domain and a tandem membrane-targeting pleckstrin homology (PH) domain. DOCK-type Rho GEFs have a membrane-targeting DHR-1 domain and a catalytic DHR-2 domain. The structures of the DH and DHR-2 catalytic domains differ, but the guanine nucleotide exchange reaction they catalyze to activate Rho GTPases is the same. Most Rho GEFs harbor additional domains that aid in their regulation. Rho GEFs adopt an auto-inhibitory conformation that is relieved by the binding of signals to their regulatory domains. DH, Dbl homology. PH, pleckstrin homology. DEP, disheveled, EGL-10 and pleckstrin. PDZ, PSD−95, DLG, ZO-1 protein–protein interactions. PEST, motif rich in proline (P), glutamic acid (E), serine (S), and threonine (T). CC, coiled coil. Ex, conserved sequence in Tiam1. RBD, Ras-binding domain. CH, calponin homology. C1, zinc finger cysteine-rich domain. SH3/2, SRC Homology 3/2. LH, Lsc homology. Sec14, lipid-binding domain. Spectrin, three-helix bundle structures. Ig, Immunoglobulin-like. FibIII, fibronectin III binding. Kinase, serine/threonine protein kinase. SMART EMBL software was used to determine domain structure.

**Figure 3:**
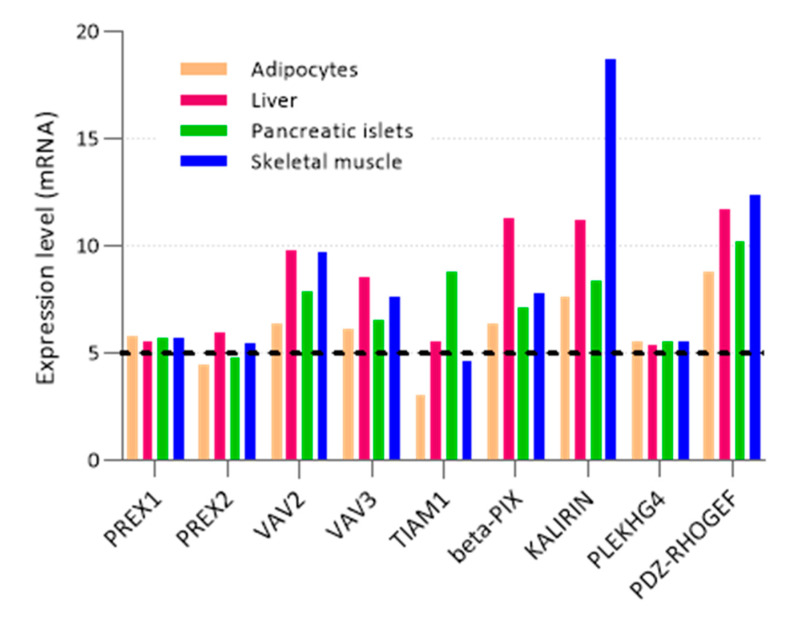
Metabolic tissue distribution of mammalian Rho GEFs that are known to be involved in the regulation of glucose homeostasis. Nine mammalian Rho GEFs are currently known to regulate glucose homeostasis in vitro or in vivo. Their distribution in human metabolic tissues is shown here, as extracted from public database BioGPS (http://biogps.org, accessed on 10 April 2021) [74]. Data are mean mRNA expression values determined by Affymetrix microarray. Units are z-scores of mean fluorescence intensity, determined using multiple probes for each transcript and processed using gcrma algorithms. Genes with z-scores above 5, indicated by the stippled black line, are considered to be expressed in that tissue. The graph was drawn using GraphPad Prism 8.

The Rho GEFs are classified into two families, Dbl and DOCK. Dbl was the first identified mammalian GEF, isolated from a human diffuse B cell lymphoma, and has become the prototype of the Dbl family [71,72]. Dbl-type GEFs have two highly conserved domains—a catalytic Dbl homology (DH) domain and a tandem membrane-targeting pleckstrin homology (PH) domain. The DH domain is necessary, and usually sufficient, for GEF activity, binding the GTPase and catalyzing the exchange of GDP for GTP in the binding pocket. The tandem PH domain is usually involved, but not sufficient, for membrane targeting, for example through binding of phosphoinositides. The DOCK family of GEFs were discovered later and are characterized by the presence of two domains: the membrane-targeting DOCK homology region-1 (DHR-1) and the catalytic DHR-2 domain [73]. DOCK GEFs can activate Rac and/or Cdc42, but not Rho [73]. Rho GEFs can often activate more than one Rho GTPase. As a general rule, Rho GEFs are regulated by auto-inhibition, which is relieved by signals that free the catalytic GEF domain to activate its target GTPase [10]. Rho GEFs are multidomain proteins, and each type is usually regulated through many different mechanisms, including complex formation with other proteins, lipid binding and/or phosphorylation. The domain structures of Rho GEFs which are known to be involved in the regulation of glucose homeostasis are shown in Figure 2. Nine of these Rho GEFs have been implicated in mammalian glucose homeostasis in vitro or in vivo, and one in yeast. The expression pattern of the nine mammalian Rho GEFs in human metabolic tissues, is shown in Figure 3. These data, extracted from public database http://biogps.org, accessed on 10 April 2021 [74], show that the Rho GEFs are expressed widely throughout major metabolic organs, with levels of Kalirin being particularly high in skeletal muscle. The data suggest that Tiam1 may not be expressed in adipose tissue and that levels of P-Rex2 in adipose tissue and pancreatic islets, or of Tiam1 in skeletal muscle, are low or absent. However, it must be considered that such mRNA data do not necessarily reflect protein levels of Rho GEFs in these tissues. It is known, for example, that P-Rex2 protein levels are much higher in the liver than in skeletal muscle of mice [75], and Tiam1 protein is expressed in skeletal muscle, [76] despite the mRNA data suggesting otherwise. The mRNA data do, however, emphasize an important gap in current understanding: the liver is a major metabolic organ that expresses multiple Rho GEFs; however, there is, to our knowledge, no research data available yet on glucoregulatory roles of Rho GEFs in this organ. More details on the expression, general functions and mechanisms of regulation of these Rho GEFs are given in Section 4, where we describe their roles in glucose homeostasis.

## 2. Glucose Homeostasis

The human body is highly dependent on the tight regulation of glucose homeostasis. Glucose is an essential metabolic source of energy, and the majority of cells require glucose for metabolic function such as respiration, protein synthesis or energy storage as glycogen. The human brain accounts for 60% of all glucose uptake from the blood, and a further 25% is taken up by the liver and gastrointestinal tissues in the unstimulated and rested state [77]. Both of these absorption processes are insulin independent. Only 25% of glucose uptake is insulin dependent, with the majority of this occurring in adipose and skeletal muscle tissues. It is vital to maintain blood glucose within its physiological range, and in the fully-grown adult human, this is between 4 and 7.8 mM or 72.0 and 140.4 mg/dL, with fasting levels at approximately 5.5 mM/99 mg/dL [78]. Blood glucose levels that differ significantly above or below this range can lead to hyper- and hypoglycemia, respectively. The clinical symptoms of these conditions can range from mild, headaches and tiredness, to severe, coma and death, so it is important to maintain the levels within a narrow range. Following a meal, food is digested and the nutrients are absorbed into the blood circulation from the intestines. Blood glucose levels transiently rise postprandial (after a meal), but return to the resting baseline level due to homeostatic mechanisms. In the fasting state, blood glucose levels are maintained through the liver by glycogenolysis, breakdown of glycogen to glucose, and gluconeogenesis, glucose synthesis from non-carbohydrate sources such as amino acids. 

The major organs involved in glucose homeostasis are the brain, digestive tract, pancreas, liver, muscle and adipose tissue. Pancreas, muscle and adipose tissue are discussed in this review, and briefly also hormonal control by the brain. The digestive tract and liver, however, will not be covered further here, as there is limited data available on the role of Rho GTPases and Rho GEFs in the glucoregulatory functions of these organs. Major glucoregulatory hormones are insulin, which triggers glucose uptake into muscle, adipose and liver cells; and glucagon, which promotes glucose liberation from glycogen in hepatocytes and de novo glucose production. Other important glucoregulatory hormones include amylin, which suppresses postprandial glucagon secretion and slows the rate of gastric emptying, and the incretins, glucose-dependent insulinotropic peptide (GIP) and glucagon-like peptide-1 (GLP-1), which are released from the gut to regulate the amount of postprandial insulin release, amongst other actions that affect blood glucose levels [79]. Among glucoregulatory hormones, insulin and the processes it regulates are the most widely studied, because dysregulation of insulin signaling and loss of insulin sensitivity lead to the development of insulin resistance, (defined here as a less-than-expected lowering of blood glucose levels in response to insulin), a hallmark of metabolic syndrome, and to type 2 diabetes [80,81]. Accordingly, the currently known roles of Rho GTPases and Rho GEFs in glucose homeostasis revolve around insulin secretion and insulin-dependent glucose uptake, so the following sections will focus on these processes.

The pancreas is a key organ involved in glucose homeostasis. As an endocrine organ, the pancreas has islets of Langerhans, a collective of several cell types that secrete hormones, including α and β cells. Glucagon is produced by the pancreatic α cells, and promotes glucose liberation from glycogen in hepatocytes and de novo glucose production. Insulin is produced by the pancreatic β cells, is released in response to high blood glucose levels, and causes glucose uptake into muscle and adipose tissue, as well as glycogen storage in the liver. Insulin secretion from pancreatic β cells is initiated by glucose transporter-mediated entry of glucose into the β cell (GLUT1/3 in humans, GLUT2 in mice) [82]. The signal is transduced into an increase in the ATP/ADP ratio and closure of the ATP-sensitive potassium channels present on the plasma membrane, leading to membrane depolarization and the influx of Ca^2+^ through voltage-gated calcium channels. The resulting rise in intracellular Ca^2+^ concentration is critical for the exocytosis of insulin granules to the plasma membrane, where fusion of the secretory granule leads to release of insulin into the blood circulation. 

Insulin-dependent glucose uptake mainly occurs in the skeletal muscle and adipose tissue. This is enabled by the insulin-stimulated translocation of the GLUT4 transporter to the plasma membrane, to allow glucose entry into the cell [83]. Insulin binds to its insulin receptor tyrosine kinase, leading to phosphorylation of insulin receptor substrate protein 1 (IRS1) and recruitment of phosphoinositide 3-kinase (PI3K), which leads to elevated levels of the lipid second messenger phosphatidylinositol (3,4,5)-trisphosphate (PIP_3_) [84]. Elevated phosphoinositide levels lead to Akt2 activation through phosphoinositide-dependent kinase (PDK)-1 and Rictor/mTORC2. Akt2 plays an important role in the maintenance of glucose homeostasis [85,86], although, there are contrasting reports indicating that a reduction in Akt activity does not lead to insulin resistance, or altered glucose uptake [87]. It is generally understood that Akt2 controls GLUT4 translocation from intracellular glucose transporter storage vesicles to the plasma membrane, through inhibiting AS160, a GAP for Rab GTPases [88,89,90,91]. A separate signaling pathway activated by insulin to induce GLUT4 translocation to the plasma membrane, involves the insulin receptor and PI3K pathway-dependent activation of the Small GTPase Rac1 [53,90,92,93,94]. This leads to actin branching and rearrangement of actin filaments [95]. It should be noted that the canonical insulin/PI3K/Akt2 signaling pathway is not restricted to muscle or adipose cells, and has pleiotropic effects in addition to GLUT4 translocation, including gene expression, cell growth, and cell survival [84,96]. An adipocyte-specific Akt2-independent pathway for glucose uptake involving the Rho GTPase TC10 has is essential for maintaining whole-body glucose homeostasis, as it accounts for the majority of insulin-stimulated also been identified, and will be discussed in the subsequent section [97]. There are also insulin-independent mechanisms of glucose uptake into skeletal muscle cells. These include muscle contraction, which promotes glucose entry by stimulating GLUT4 translocation, and constitutive glucose uptake through GLUT1. 

## 3. Rho GTPases in Glucose Homeostasis

The control of glucose homeostasis by Rho family GTPases is an emerging field. Best understood are the roles of Rac1, Cdc42 and RhoA in insulin-dependent glucose uptake into adipose and skeletal tissues, and the roles of Rac1 and Cdc42 in glucose-stimulated insulin secretion by pancreatic β cells. There is already an extensive, recent review by Møller et al. on the involvement of Rho GTPases in these processes [98]. This excellent review enabled us to keep the subsequent section brief, we will summarize key findings and update on new literature. The main body of our review will focus instead on Rho GEFs, which were not covered by Møller et al.

Both Rac1 and Cdc42 have been implicated in glucose-stimulated insulin secretion by pancreatic β cells via their roles in actin rearrangement [99,100,101,102]. During the fasting state when blood glucose levels are low, the actin cytoskeleton of pancreatic β cells prevents insulin storage vesicles from fusing with the plasma membrane and releasing insulin. An increase in blood glucose levels leads to actin cytoskeleton rearrangements, to allow granule fusion and thus insulin release. This granule translocation process has been shown to be dependent on both Cdc42 [102] and Rac1 [99] activation, and is likely mediated through the Rac effector PAK1 [50,103], although the precise mechanism is still unclear. 

Skeletal muscle is essential for maintaining whole-body glucose homeostasis, as it accounts for the majority of insulin-stimulated glucose uptake [104,105]. Insulin activates Rac1 and RhoA in muscle cells [92,94,106]. It is accepted that actin remodeling by Rac1 is required for GLUT4 translocation to the sarcolemma [53,92,107]. Insulin-stimulated GLUT4 translocation allows glucose entry into skeletal muscle cells. Accordingly, many studies have identified Rac1 as an essential regulator of insulin-dependent glucose uptake into skeletal muscle [92,94,107,108]. These include genetic studies using tissue-specific inducible knockout of Rac1 in the skeletal muscle of mice [53]. 

Rac1 has also been implicated in insulin-independent mechanisms of glucose uptake in muscle cells. Sylow et al. found that Rac1 is activated in both mouse and human skeletal cells following muscle contraction induced by physical exercise. Muscle-specific inducible Rac1 deficiency in mice, or pharmacological inhibition of Rac1 with NSC23766 or a derivative of this compound decreased the contraction-stimulated glucose uptake [109]. AMP-activated protein kinase (AMPK) and Rac1 pathways were found to be important for the regulation of contraction-induced muscle glucose uptake ex vivo, in the muscle-specific AMPK β1β2 KO mouse [110]. The Rac1 inhibitor II and deletion of AMPK β1β2, independently, and additively, led to decreased 2-deoxy-D-glucose transport in soleus muscle ex vivo. Yet, when the authors investigated this in vivo using a muscle-specific kinase-dead AMPK mouse and inducible muscle-specific Rac1 KO mouse, they found that exercise-induced glucose uptake depends on Rac1 and not AMPK in mice [110]. These differences highlight the importance of in vivo experiments. Rac1 has also been shown to regulate exercise-induced GLUT4 translocation to the plasma membrane of skeletal muscle cells in vivo [111]. The authors visualized GLUT4 translocation by immunohistochemistry of cryosections from the tibialis anterior muscle of muscle-specific Rac1 KO mice, and found it to be reduced compared to wild type.

The role which RhoA plays in regulating insulin-stimulated glucose uptake in skeletal muscle is more widely contested, due to lack of data and indirect, inconsistent evidence [98]; however, the involvement of the downstream RhoA effector ROCK is likely [112,113]. There are two ROCK isoforms, ROCK1 and ROCK2, and these are likely to have different effects on glucose uptake, due to differences in actin regulation mechanisms, subcellular distributions, and interactions with IRS1 [114]. Downregulation of ROCK1 in 3T3-L1 adipocytes and L6 myoblasts decreased insulin-stimulated glucose transport in a manner dependent on actin cytoskeleton remodeling [114]. The role of RhoA in glucose transport in L6 myocytes was examined by siRNA knockdown [115]. RhoA knockdown attenuated glucose transport, whereas RhoA overexpression increased basal, but not insulin-induced glucose uptake. The authors found that RhoA signals through an Akt-independent mechanism, where in the starved state (absence of insulin), Akt signaling was minimal. RhoA was found to elicit its effects on the actin cytoskeleton by the ROCK pathway and ezrin/radixin/moesin proteins [115]. 

Adipose tissue is essential for maintaining glucose homeostasis, and for the postprandial reduction in blood glucose levels. Rho GTPases play similar roles in adipose tissue, as in skeletal muscle, regulating glucose uptake through translocation of GLUT4. The literature is less extensive and more controversial, but overall glucose homeostasis in adipose tissue is thought to be regulated by different combinations of Rho GTPase family members compared to muscle cells [92]. In adipocytes, insulin stimulation leads to the activation of Rho GTPases TC10, Rac1, Cdc42 and RhoA [97,116,117,118]. Cdc42 and the Cdc42-like TC10 are the most studied Rho family GTPases in adipocyte metabolism. siRNA knockdown of Cdc42 in 3T3-L1 adipocytes confirmed Cdc42 as a regulator of insulin-mediated GLUT4 translocation [116]. TC10 regulates actin rearrangement likely through N-WASP and is important for insulin-induced GLUT4 translocation, as dominant-negative TC10 (T31N) prevents F-actin formation, GLUT4 translocation and subsequent glucose transport [119]. TC10 is activated in response to insulin, mediated by translocation of the E3 ubiquitin ligase Cbl to lipid rafts and recruitment of C3G, a GEF for Rap-family GTPases, which indirectly leads to TC10 activation [97]. The downstream effector of TC10 in actin cytoskeleton rearrangement, Cdc42 interacting protein-4 (CIP4), is also required for insulin-stimulated GLUT4 translocation in adipocytes [120]. 

RhoA was identified to be activated by insulin and to induce GLUT4 translocation and glucose uptake in a transfection study in rat adipocytes [121]. Other studies contested this role of RhoA, as expression of dominant-negative or constitutively active mutants of RhoA in 3T3-L1 adipocytes had no effect on insulin-stimulated GLUT4 translocation and glucose uptake [97]. A recent study has gone further to differentiate between the different isotypes of Rho. The authors found that RhoA, but not RhoB or RhoC controls glucose transport in adipocytes, through regulation of the actin cytoskeleton [115]. 

The role of Rac1 in adipose tissue is unclear, and some have suggested Rac1 does not regulate insulin-induced glucose uptake in adipocytes [122]. A recent study by Takenaka et al. [123] contests the observation made by Marcusohn et al. that neither dominant-negative nor constitutively active ectopically expressed Rac1 mutants affected glucose uptake in 3T3-L1 adipocytes [122]. Takenaka et al. used the Rac1 inhibitor II (CAS 1090893-12-1); to suppress GLUT4 translocation induced by insulin or by constitutively active mutants of Akt2 or PI3K in L1-GLUT4 adipocytes [123]. Unlike Marcusohn et al., the authors also saw enhanced GLUT4 translocation in response to constitutively active Rac1. The exact role of Rac1 in adipocyte glucose homeostasis, if any, requires further study.

## 4. Rho GEFs in Glucose Homeostasis

Recent research efforts have begun to decipher which Rho GEFs are responsible for the activation of Rho family GTPases in glucose homeostasis. However, considering the large number of Rho GEFs and processes that have not yet been investigated, it is clear that much remains to be explored. Currently best understood are the roles of Rho GEFs in the translocation of GLUT4 glucose transporter and glucose-stimulated insulin uptake in adipose and skeletal tissue, and in the glucose-stimulated secretion of insulin by pancreatic β cells. Mouse models have been more widely used to study the roles of Rho GEFs than Rho GTPases, and this approach has shown that Rho GEFs are important regulators of glucose homeostasis in vivo, with deficiencies or deregulated expression commonly resulting in impaired insulin signaling, insulin resistance, glucose intolerance, and the development of metabolic syndrome and type 2 diabetes. We will discuss these findings here for individual Rho GEFs.

### 4.1. P-Rex

**Figure 4:**
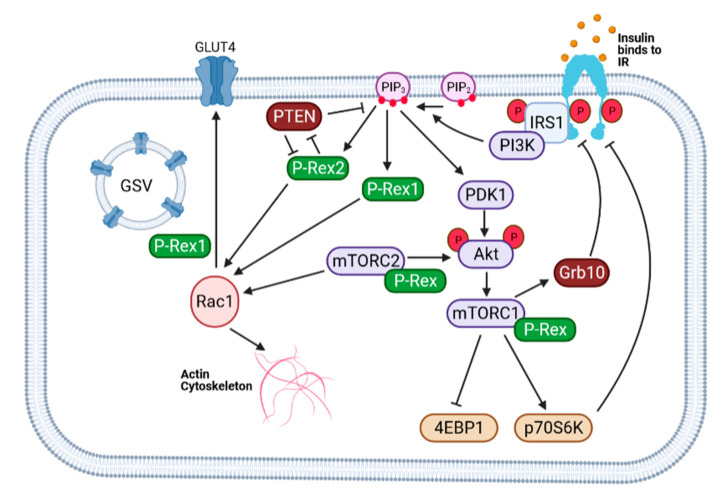
P-Rex family Rac GEFs in insulin signaling. Insulin activates the insulin receptor (IR), leading to receptor conformational changes which promote transphosphorylation and subsequent activation of the receptor tyrosine kinase activity. Insulin receptor substrate-1 (IRS1) is phosphorylated and recruits PI3K to the plasma membrane. Class 1 PI3Ks phosphorylate PtdIns(4,5)P_2_ to generate PIP_3_ [84]. PIP_3_ promotes the translocation of PDK1 and Akt to the membrane through interaction with the PH domains, and phosphorylation of Akt by PDK1 and mTORC2 (PDK2) leads to the full activation of Akt. P-Rex1 and P-Rex2 are direct binding partners of mTORC1 and mTORC2 [132], both of which are important effectors of the insulin-signaling pathway. Both P-Rex proteins activate Rac1, and P-Rex1 has been implicated in the insulin-dependent translocation of GLUT4 to the plasma membrane in adipocytes [137]. P-Rex2 has been identified to inhibit PTEN through a mechanism independent of the GEF activity, and thus regulates the PIP_3_ signal [129,130]. Inversely, PTEN also inhibits P-Rex2 [131]. Figure made with BioRender.

The P-Rex family proteins, P-Rex1 and P-Rex2, are Rac GEFs for Rac proteins (Rac1, Rac2, Rac2 and RhoG) [124,125,126]. The human *PREX1* gene maps to a gene locus on chromosome 20q13.1 that has been linked to type 2 diabetes in many studies [127], and SNPs in the perigenic region of *PREX1* have been proposed to associate with the likelihood of obesity developing into type 2 diabetes [128]. P-Rex1 is highly expressed in leukocytes and neurons, but is present at lower levels in many other cell types, whereas P-Rex2 is more widely expressed except in leukocytes. P-Rex-deficient mouse strains have revealed roles for P-Rex1 in the pro-inflammatory functions of leukocytes and developmental migration of melanocytes, and roles for P-Rex2 in neuronal morphology and plasticity, and in motor coordination [124]. Both P-Rex proteins are activated in response to the stimulation of G protein-coupled receptors (GPCRs), by the Gβγ subunits of heterotrimeric G proteins, and in response to the stimulation of PI3K-coupled receptors, by PIP_3_, with additional regulation through phosphorylation and protein /protein interactions [124]. P-Rex family Rac GEFs are known to facilitate insulin signaling [124]. Upon insulin stimulation, P-Rex1 responds to the activation of PI3K and the production of PIP_3_, and transmits the insulin signal through its catalytic Rac GEF activity. P-Rex2 may have this same role, but has additionally been identified to inhibit the tumor suppressor PTEN. PTEN metabolizes PIP_3_ to terminate PI3K signaling, and thus the P-Rex2-mediated inhibition of PTEN promotes PI3K signaling pathways such as Akt activation [129,130]. The inhibition of PTEN is independent of Rac-GEF activity, mediated through the PH domain of P-Rex2 binding to the catalytic and C2 domains of PTEN, and the IP4P domain of P-Rex2 contacting the C-terminal PDZ-binding domain of PTEN. There is also, inversely, an inhibition of P-Rex2 Rac GEF activity by PTEN through a phosphatase-independent mechanism [131]. It has been proposed that P-Rex2-mediated insulin signaling may depend on PTEN inhibition rather than its Rac GEF catalytic activity, but this remains to be investigated. The roles which P-Rex family GEFs play in insulin signaling and the pathways linking them together are summarized in Figure 4.

An important binding partner of P-Rex proteins is the kinase mammalian target of rapamycin (mTOR), which they bind constitutively through their DEP domains. As such, P-Rex Rac GEFs form part of both the mTORC1 and mTORC2 protein complexes [132]. mTORC1 is a central regulator of protein synthesis, cell growth and cell metabolism, involved in signaling pathways that control glucose metabolism and the synthesis of nucleotides and lipids, through many downstream effectors [133]. For example, mTORC1 facilitates cell growth by promoting glycolysis through an increase in the translation of the transcription factor HIF1α, leading to the expression of glycolytic enzymes [133]. mTORC2 was first recognized for its contribution to Rho GTPase activation and cytoskeletal rearrangements, but it also induces the full activation of Akt by acting as PDK2. mTORC2 has been shown to the regulate whole-body glucose metabolism in the mouse. For example, a fat cell-specific knockout of the mTORC2 component Rictor prevented insulin-stimulated phosphorylation of Akt, and subsequent phosphorylation of downstream targets such as AS160 [134], a Rab GAP involved in the translocation of GLUT4 to the plasma membrane [135]. mTORC2 may also play a role in muscle insulin resistance [136]. Kleinert et al. showed that mTORC2 inhibition by mTOR kinase inhibitor AZD8055 decreased insulin-stimulated glucose uptake into L6 muscle cells, but this was not due to alterations in GLUT4 translocation in L6-GLUT4myc myoblasts. Instead, mTORC2 inhibition induced a defect in glycolysis, affecting the concentration gradient required for glucose uptake, but this was independent of Akt activity. ADZ8055 induced insulin resistance in wild-type mice in vivo, characterized by elevated blood glucose levels after feeding, compared to control mice without the drug. Glucose uptake was also impaired in muscle-specific Rictor knockout (a subunit of mTORC2) muscle cells in vivo. The functional consequences of the interaction of P-Rex Rac GEFs with mTORC1 and mTORC2, however, remain to be investigated, particularly in the context of glucose homeostasis.

#### 4.1.1. P-Rex1 in Pancreatic β Cells 

**Figure 5:**
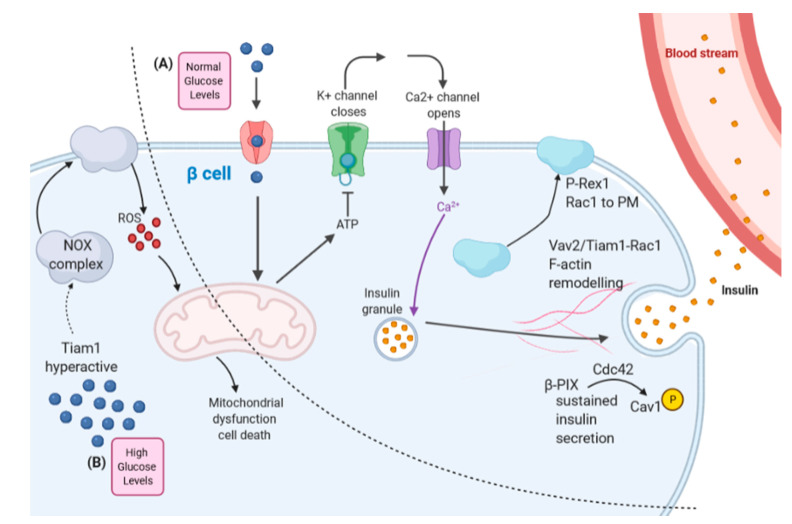
The roles of Rho family GEFs in pancreatic β cell glucose homeostasis. (**A**,**B**) depict normal and un-physiologically high glucose conditions, respectively, separated by the dashed line in the figure. (**A**) Under conditions of normal glucose homeostasis in the pancreatic β cell, glucose entry mediates insulin secretion. Vav2 and Tiam1-Rac1 have been implicated in remodeling the F-actin cytoskeleton to enable this secretory process [42,98]. P-Rex1 was implicated in glucose-induced insulin secretion and shown to aid in Rac1 localization at the plasma membrane (PM) under high-glucose conditions [77]. β-PIX competes with caveolin-1 (Cav1) for binding to Cdc42 and activates Cdc42 as part of the secondary sustained phase of insulin secretion. (**B**) Stress-related levels of glucose (or fatty acids) lead to the apoptotic death of pancreatic β cells and a diabetic phenotype, and this is possibly linked to hyper-activation of the Tiam1–Rac1 pathway. Figure made with BioRender.

Recently, new data have emerged indicating a role of P-Rex1 in glucose-stimulated insulin release from pancreatic β cells [138]. siRNA knockdown of P-Rex1 in the rat insulinoma INS-1 832/13 pancreatic β cell line led to a decrease in glucose-stimulated insulin secretion, and inhibition of glucose-stimulated Rac1 activation. Knockdown of P-Rex1 also reduced the membrane association of Rac1 under high glucose conditions (Figure 5). Rac can usually translocate to the plasma membrane independently of its GEFs, upon release from Rho-GDI, although activation by GEFs contributes to retaining the GTPase at the membrane [139,140]. It would be of interest therefore, to investigate if this phenotype can be rescued by reintroduction of a catalytically inactive P-Rex1 protein. The authors also investigated the role of RhoG in glucose-stimulated insulin secretion, as P-Rex1 has GEF activity towards RhoG as well as Rac1, but no contribution of RhoG was found, suggesting that P-Rex1 may regulate this process through its Rac1 GEF activity. Future in vivo work is needed using the P-Rex1-deficient mouse model, and ex vivo analysis of islets of Langerhans, to investigate the role of P-Rex1 in glucose-stimulated insulin secretion.

#### 4.1.2. P-Rex1 in Adipocytes 

Overexpression of P-Rex1 has been shown to regulate the insulin-dependent trafficking of GLUT4 in 3T3-L1 adipocytes [137]. This is conferred through its catalytic GEF activity towards Rac1, leading to actin remodeling in membrane ruffles and PIP_3_ formation. siRNA-mediated knockdown of P-Rex1 reduced insulin-stimulated glucose uptake, supporting a role for the Rac GEF in this process [137]. In light of these findings, future studies should seek to determine whether the P-Rex1-deficient mouse shows an insulin-resistant phenotype. 

P-Rex1 was shown to control metabolism also by conferring the thermogenic capacity of brown adipose cells [141]. Xue et al. used microarray analysis of immortalized human adipose tissues to investigate thermogenic potential. P-Rex1 was identified as a positive UCP1 regulator, and CRISPR-Cas9 was used to knock out P-Rex1 in brown pre-adipocytes. P-Rex1 deficiency did not affect the ability of pre-adipocytes to differentiate into adipocytes, but did abolish UCP1 levels, respiration capacity and proton leak in the mature brown adipocytes. This suggests an important role for P-Rex1 in the thermogenic potential of brown adipose tissue.

#### 4.1.3. P-Rex2 In Vivo

An investigation into the role of P-Rex2 in insulin signaling has brought to light that P-Rex2 affects glucose homeostasis in vivo. Hodakoski et al. suggested that whole-body P-Rex2 deficiency in mice causes insulin resistance [129]. After insulin injection, the blood glucose levels of P-Rex2-deficient mice did not remain low for as long as in wild-type mice, although the initial drop in blood glucose levels was normal. This would suggest that P-Rex2 deficiency may not cause bona fide insulin resistance, but rather affects the maintenance of the insulin response. Further investigation showed reduced Akt phosphorylation in adipose tissue after insulin injection. The authors also showed that P-Rex2-deficient mice were glucose intolerant in vivo, as these mice failed to clear glucose from the bloodstream after glucose challenge at the same rate as wild-type mice. Finally, insulin-resistant humans were also shown to have lower levels of P-Rex2 protein in insulin-sensitive tissues, such as adipose tissue [129]. 

As described above, P-Rex2 inhibits the tumor suppressor PTEN, leading to elevated levels of PIP_3_ and PI3K signaling pathway activity [129]. On this basis, Hodakoski et al. proposed that the role of P-Rex2 in glucose homeostasis may be mediated through PTEN inhibition rather than the Rac GEF catalytic activity. The crystal structure of human P-Rex1 has been solved and confirmed the key residues for the catalytic Rac GEF activity [142,143]. Future studies using P-Rex1 and P-Rex2 mouse models that lack Rac GEF catalytic activity should seek to determine whether these metabolic phenotypes are due to canonical GEF activity or adapter function such as the P-Rex2-mediated inhibition of PTEN. 

### 4.2. Vav 

The Vav family GEFs are Dbl-type GEFs, and there are three members in mammalian cells [3]. Vav1 is mainly restricted to the hematopoietic system, whereas Vav3 has a broader expression profile and Vav2 is almost ubiquitously expressed. The Vav proteins are GEFs for Rac1 and, to a lesser extent, RhoA. Their catalytic activities are stimulated by tyrosine phosphorylation and modulated by a range of other signals, including phosphoinositides. The classical known functions of Vav proteins have been discovered in mice. Vav2 is important for B cell maturation [144] and blood vessel relaxation induced by nitric oxide [145]; Vav3 plays roles in the nervous system including cerebellar development [146]; and Vav1 is important for many immune cell responses [147]. 

#### 4.2.1. Vav2 In Vivo

A study by Menacho-Márquez et al. from 2013 using mice with whole-body Vav2 deficiency showed that these mice did not have glucose intolerance, hyperglycemia or liver steatosis, and suggested that Vav2 does not play a significant role in metabolic regulation [148]. However, recently, a role for involvement of Vav2 in metabolic regulation has been revealed. Rodríguez-Fdez et al. found that mice with low catalytic activity of Vav2 were predisposed to high-fat diet-induced metabolic imbalance, including reduced Akt and Rac1 signaling in insulin-stimulated skeletal muscle, but not in adipose tissue or the liver [149]. These mice exhibited reduced skeletal muscle mass and decreased responsiveness to insulin, leading to a metabolic syndrome-like condition. This phenotype was reversed in mice expressing Vav2 with catalytic hyperactivity, as these mice were protected from the high-fat diet-induced metabolic imbalance [149]. The authors went on to investigate Vav2-dependent responses to insulin in murine C2C12 myoblast cells, and showed these to be mediated by the activation of Rac1 and the downstream activation of PI3K and Akt. Vav2 depletion delayed the translocation of GLUT4 to the plasma membrane [149]. The difference in phenotype between mice with whole-body deletion of Vav2 and altered Vav2 catalytic activity exemplifies the importance of sophisticated mouse models for evaluating potential compensation or redundant functions with other GEFs, and for the evaluation of GEF activity dependent effects compared to adaptor functions.

#### 4.2.2. Vav2 in Pancreatic β Cells 

A study by Veluthakal et al. highlighted Vav2 as a GEF for Rac1 in glucose-stimulated insulin secretion, through the use of Vav2 siRNA knockdown in INS-1 832/13 pancreatic β cells [101]. Using the Vav2:Rac1 interaction inhibitor EHop−016, and live cell imaging with Lifeact-GFP biosensor, the authors were able to show a marked reduction in F-actin depolymerization during the second phase of insulin secretion. This phase requires F-actin cytoskeleton remodeling to allow the movement of granules from intracellular to plasma membrane localization (Figure 5). It should be noted, however, that EHop−016 is unlikely to be a specific inhibitor of Vav2, as it was designed to block the binding of Rho GEFs to Rac [150], and as this review shows, other Rho GEFs are also implicated in the actin remodeling processes associated with insulin secretion.

#### 4.2.3. Vav3 In Vivo

Menacho-Márquez et al. found that whole-body deficiency in Vav3 leads to varying metabolic phenotypes depending on the type of diet. Vav3-deficient mice on chow diet developed metabolic syndrome, non-alcoholic fatty liver disease (NAFLD) and type 2 diabetes, but not increased adiposity [148]. Conversely, Vav3-deficient mice on high-fat diet were protected from obesity and metabolic syndrome due to increased levels of energy consumption per lean mass and brown adipose tissue thermogenesis. These complex diet-dependent effects were traced back to the abnormal regulation of the sympathetic nervous system due to loss of Vav3, which led to enhanced white-to-brown adipose transdifferentiation. 

#### 4.2.4. Vav3 in Skeletal Muscle Cells

Vav3 has also been shown to be involved in metformin-mediated glucose uptake. Metformin is a drug used to treat diabetes, as it increases the effects of insulin through suppression of gluconeogenesis and glycogenolysis in the liver, and the stimulation of insulin signaling and glucose transport into muscles [151,152,153]. One of the mechanisms of action of metformin is through activation of AMPK [154,155]. Vav3 expression in C2C12 myoblast cells rose under high glucose conditions as a consequence of AMPK signaling. Knockdown of Vav3 in these cells potentiated metformin-mediated GLUT4 translocation and glucose uptake [156].

### 4.3. Tiam

The Tiam (T cell lymphoma invasion and metastasis) family of GEFs are Dbl-type GEFs specific for Rac1. There are two homologues, Tiam1 and Tiam2/STEF, which have differential upstream interactors and subcellular localizations, leading to diversification in their functions [157]. Tiam1 is the only family member currently implicated in glucose homeostasis. Tiam1 is auto-inhibited by intramolecular interactions and is largely cytoplasmic when inactive. Tiam1 translocates to the plasma membrane upon binding PIP_3_ through its N-terminal PH domain, and it contains a myristoylation sequence which provides a lipid anchor, although that alone is not sufficient to target Tiam1 to the membrane. The binding of GTP-loaded Ras GTPase to the Ras-binding domain of Tiam1 stimulates Rac1 GEF activity. Tiam1 is also regulated through phosphorylation by various kinases, through binding of the phosphoinositide PI (3)P to its C-terminal PH domain, and through protein–protein interactions. The known roles of Tiam1 are diverse. Tiam1 has been shown to play contradictory roles in cell migration and adhesion, dependent on cell type and condition, and its expression is dysregulated in many cancers [157,158]. 

#### 4.3.1. Tiam1 in Pancreatic β Cells 

The Tiam1/Rac1 signaling pathway also plays a role in glucose-stimulated insulin secretion in pancreatic β cells, through remodeling the actin cytoskeleton (Figure 5). Veluthakal et al. showed this through the use of siRNA-mediated knockdown and the Rac inhibitor NSC23766, both of which attenuated glucose-induced insulin secretion from granules in INS 832/13 cells and in rat islets of Langerhans [159]. Syed et al. proposed that hyperactivation of Tiam1/Rac1 may bring about apoptotic death of pancreatic β cells, as NSC23766 treatment reduced the superoxide production and mitochondrial dysfunction brought about by stress-related levels of glucose and saturated fatty acids [160]. The potential protective role that inhibition of Tiam1/Rac1 might play, was further shown through use of an in vivo mouse model of type 1 diabetes (NOD mouse), where intraperitoneal daily injection of NSC23766 significantly prevented the development of spontaneous diabetes [161]. Furthermore, NSC23766 was used to implicate Tiam1/Rac1 in the development of diabetic retinopathy, through the activation of Nox2 and p38 MAP kinase leading to mitochondrial dysfunction and retinal endothelial cell death [162,163,164]. It should be noted that NSC23766 is still widely used as a Rac inhibitor, and was originally proposed to block the Tiam1-mediated activation of Rac1 [165]. The limited efficacy and selectivity of this inhibitor mean conclusions regarding the involvement of specific Rac GEFs must backed up by other means. Overall, the role of Tiam1 in pancreatic cells needs to investigated further by in vivo studies in Tiam1-deficient mice.

#### 4.3.2. Tiam1 in Skeletal Muscle Cells 

Tiam1 has been identified to play a key role in metformin-mediated glucose uptake into skeletal muscle cells. You et al. investigated glucose uptake in C2C12 myoblast cells and showed that metformin induces an AMPK-dependent interaction between Tiam1 and the scaffold protein 14:3:3, and that Tiam1 is required for metformin-mediated GLUT4 translocation to the plasma membrane [166]. This study did not investigate the physiological consequences of Tiam1-mediated GLUT4 translocation, so further studies should be performed in vivo. The authors did, however, use the hyperglycemic db/db mouse model, in which they found downregulated Tiam1 mRNA levels in quadriceps muscle compared to wild type. This corroborated cell culture data showing that high glucose conditions lead to downregulation of Tiam1, indicating a role both in vitro and in vivo for Tiam1 in diabetes. In a different study, Tiam1 has also been reported to induce GLUT4 translocation in muscle cells [167]. Overexpression of Tiam1 stimulated the translocation of GLUT4 to the membrane of L6 GLUT4myc muscle cells, and led to F-actin-rich membrane ruffles, indicative of endogenous Rac1 activation. 

Tiam1 has also been implicated in contraction-stimulated glucose uptake in skeletal muscle cells. Contraction-dependent glucose uptake into skeletal muscle is known to require AMPK and the Rab-GAPs AS160 and TBC1D1; however, as described above, more recently, Rac1 has also been associated with this response [110,111]. AMPK activity lies upstream of Rac1 activation during the electric pulse-stimulated contraction of C2C12 myotubes, as well as during contraction of the gastrocnemius muscle in mice [168]. In vitro siRNA knockdown of Tiam1 in differentiated C2C12 myotubes inhibited Rac1 activation by electrical pulse-stimulation [76]. Electric pulse-stimulated glucose uptake, as well as the expression and translocation of GLUT4 were inhibited by Tiam1 knockdown. Finally, Tiam1 protein levels in mouse gastrocnemius muscle were elevated upon exercising of wild-type mice on a treadmill [76]. 

### 4.4. β-PIX

β-PIX (PAK-interacting exchange factor) is a Rac, Cdc42 and TC10-specific Dbl-type GEF which binds to and is phosphorylated by PAK [47]. β-PIX is regulated through phosphorylation by Ptk2/Fak1, which promotes the interaction with Rac1, and through phosphorylation by CaMK1, which enhances the GEF activity. β-PIX has been implicated in the regulation of focal adhesion maturation [169] and in actin and membrane remodeling [170], as well as in regulating the transcription of β-catenin [171], and it is essential for axon formation during cortical development [172]. 

#### β-PIX in Pancreatic β cells 

Kepner et al. identified β-PIX as a Cdc42 GEF in MIN6 pancreatic β cells [173]. siRNA-mediated knockdown of β-PIX reduced glucose-stimulated insulin secretion in these cells. Cdc42 is a key regulator of the secondary sustained phase of insulin release from granules, and leads to PAK, then Rac1 activation. Kepner et al. showed that caveolin-1, the main component of caveolae in the plasma membrane, binds Cdc42 in MIN6 β cells. They showed that β-PIX competes with caveolin-1 for Cdc42 binding at the boundary between insulin storage granule and plasma membrane. More work is needed to identify the exact role of β-PIX in the second phase insulin secretion, for example through the study of glucose-stimulated insulin secretion in β-PIX knockout mouse models or perfusion experiments ex vivo using isolated pancreatic islets.

### 4.5. Kalirin

Kalirin is a Rho GEF with two catalytic GEF domains, one with activity towards Rac1 and the other towards RhoA. Kalirin is best known for its roles in neuronal development and axonal outgrowth [174,175] and is highly expressed in the brain/CNS [176], but is also found in non-neuronal systems such as the heart, endocrine and skeletal muscle [177]. Several splice variants of Kalirin exist with different functions in neurons. The GEF also has a ubiquitously expressed homologue, Trio, but only Kalirin has been implicated in glucose metabolism to date. Uniquely among Rho GEFs, Kalirin and Trio harbor a protein kinase domain as well as their GEF activities. Membrane localization of Kalirin is mediated by Arf6, a member of the Arf family of GTPases that crosstalks with Rac1 in several pathways [178]. The catalytic activities of Kalirin are regulated by phosphorylation, for example by CamKII, and by protein–protein interactions. 

#### Kalirin in Skeletal Muscle Cells 

Kalirin plays a role in glucose uptake in skeletal muscle cells stimulated with the myokine Follistatin-like 1 (FSTL-1). Low levels of FSTL-1 are associated with diabetes mellitus, making this an important area of research [179]. FSTL-1 treatment induced the expression of Kalirin in L6 rat skeletal muscle cells [180]. A role for this GEF in FSTL-1-mediated GLUT4 translocation and glucose uptake was confirmed using siRNA knockdown. The authors also used knockdown of Kalirin in primary myoblasts to show that FSTL-1-induced glucose uptake is dependent on Kalirin-1/Rac1 in a more physiologically relevant system. The true physiological significance of this is yet to be determined, as the concentrations of FSTL-1 used were 11–200-fold higher than have been found in vivo [181]. 

### 4.6. Plekhg4 

Plekhg4, also known as FLJ00068 or puratrophin-1, is a Dbl-type Rho GEF for Rac1, Cdc42 and RhoA [182] and is regulated by ubiquitination. Plekhg4 is widely expressed, including in many epithelial cell types as well as pancreas, muscle tissue and Purkinje cells [108,183]. Plekhg4 is regulated through auto-inhibition by the interaction of its N-terminus with the C terminus, where the catalytic DH/PH domain tandem is found, and N-terminal deletion renders the GEF constitutively active [108]. Plekhg4 was originally identified through its role in the pathology of autosomal dominant cerebellar ataxia (ADCA) in a Japanese population [183]. This pathology was caused by a single nucleotide substitution in the 5′ untranslated region, and led to protein aggregation [183]. However, mutations in Plekhg4 were not found to be a common cause of ADCA in Caucasian populations [184]. Interesting new roles are emerging for Plekhg4 in glucose homeostasis.

#### 4.6.1. Plekhg4 in Skeletal Muscle Cells 

Ueda et al., identified Plekhg4 as a GEF responsible for Rac1 activation in GLUT4 translocation in muscle cells [108,185]. Several Dbl-type Rac1 GEFs that are significantly expressed in skeletal muscle were ectopically expressed in the L6-GLUT4 myoblast cell line, and only Plekhg4, amongst Dbl-I, α-PIX β-PIX, Swap70 (switch-associated protein 70) and Vav2, enhanced insulin-stimulated Rac1 activation and insulin-dependent GLUT4 translocation to the plasma membrane. Expression of constitutively active Plekhg4 and siRNA knockdown led to increased and decreased GLUT4 translocation, respectively. 

#### 4.6.2. Plekhg4 in Adipocytes

The Satoh group have shown a role for Plekhg4 in GLUT4 translocation in adipocytes [186]. Knockdown of Plekhg4 by siRNA treatment in differentiated 3T3-L1 adipocytes reduced insulin-stimulated Rac1 activity, even in the presence of constitutively active Akt2, which would usually increase Rac1 activity during the process of GLUT4 translocation. This study suggested that Plekhg4 may lie downstream of Akt2 [186]; however, this is likely to be through an intermediator protein as the GEF harbors no consensus sequence for phosphorylation by Akt. Research is required to help decipher the mechanisms that regulate Plekhg4, to enable GLUT4 translocation in skeletal muscle cells and adipocytes.

### 4.7. PDZ-RhoGEF 

Rho guanine nucleotide exchange factor 11 (PDZ-RhoGEF) is a ubiquitously expressed Dbl-type GEF that activates RhoA and is best known for its role in neurotrophin-induced neurite outgrowth [187,188]. Alongside the DH/PH domain tandem, PDZ-RhoGEF harbors a PDZ protein–protein interaction domain and an Lsc homology (LH) domain which is distantly related to Regulator of G Protein Signaling (RGS) domains, and binds to activated Gα_12/13_ family heterotrimeric G proteins (Figure 2). The presence of the LH domain links Gα_12/13_-coupled GPCRs to Rho activation. Several studies (referenced in [189]) found that single nucleotide polymorphisms in the PDZ-RhoGEF gene are associated with insulin resistance and type 2 diabetes in diverse human populations.

#### PDZ-RhoGEF In Vivo 

Chang et al. found that mice with whole-body deficiency in PDZ-RhoGEF had increased energy expenditure, as measured by an increased oxygen consumption rate in the epididymal white adipose tissue. These mice were protected from high-fat diet-induced development of insulin resistance, as their blood glucose levels dropped lower than in wild-type mice following insulin injection. The mice were also protected from developing glucose intolerance and eventually, type 2 diabetes [189]. Mechanistically, the protection from insulin resistance was found to be due to reduced p70^S6K^ signaling in white adipose tissue of PDZ-RhoGEF KO mice, and altered RhoA/ROCK-dependent phosphorylation of IRS1. In wild-type mice, high-fat diet led to elevated protein levels of PDZ-RhoGEF in insulin target tissues, as well as increased p70^S6K^ signaling and IRS1 inhibition. PDZ-RhoGEF plays a functional role in glucose homeostasis, particularly in adipose tissue. Ageing PDZ-RhoGEF-deficient mice weighed less than wild-type mice, primarily due to a reduction in white adipose tissue mass (reduced mature adipocyte number, not cell size) [189]. Adipocyte number is a factor relating to the fat mass in humans, as individuals who have higher numbers of adipocytes tend towards obesity [190]. 

### 4.8. DOCK1

Rho protein signaling in glucose homeostasis is not restricted to mammalian systems. Related processes have been found in *Saccharomyces cerevisiae* yeast. Mammalian Rac1 has a yeast homologue, Rho5, which is activated by Dck1, the yeast homologue of mammalian DOCK1 [191]. DOCK1, also known as DOCK180, is the founding member of the DOCK-type Rho GEFs, which are characterized by their lipid-binding DOCK homology region-1 (DHR-1) domain and catalytic DHR-2 GEF domain (Figure 2). DOCK1 functions by binding Elmo1 through the SH3 domain, which induces membrane translocation of DOCK1 and conformational change that activates the GEF. In yeast, Dck1 functions equivalently in conjunction with Lmo1. 

#### DOCK1 in the Yeast Stress Response 

There are data suggesting that Rho5 may be implicated in the stress response of yeast cells to low glucose conditions [192]. Glucose starvation induces Rho5 relocation from the plasma membrane to the mitochondria, and this translocation is conferred by Dck1/Lmo1, implicating the GEF in the change from cytoplasmic glycolytic fermentation to oxidative phosphorylation where energy production is more efficient. It is unknown whether this translates to mammalian cells, as there are key differences between the main roles of mammalian Rac1 and yeast Rho5. For example, Rac1 in mammals primarily controls actin dynamics. However, this role is only weakly conserved in the yeast homologue [192]. 

### 4.9. Summary of Rho GEF Functions in Glucose Homeostasis

In summary, 10 different Rho GEFs have been implicated to date in the regulation of glucose homeostasis, nine of which are in mammals, and one, the DOCK1 homologue Dck1, is in yeast. Among the mammalian Rho GEFs, P-Rex1, Vav2, Vav3, Tiam1, Kalirin and Plekhg4 have been identified in GLUT4 translocation and/or insulin-stimulated glucose uptake in skeletal muscle or adipose tissue. The Rho GEFs P-Rex1, Vav2, Tiam1 and β-PIX have been implicated in the glucose-stimulated release of insulin by pancreatic β cells. In vivo studies in mice have shown the Rho GEFs P-Rex2, Vav2, Vav3 and PDZ-RhoGEF to be involved in glucose tolerance and/or insulin sensitivity, with deletion of these GEFs either contributing to the development of metabolic syndrome or protecting mice from this disease.

## 5. Conclusions and Future Challenges 

We have summarized evidence for the emerging roles of Rho GTPases and Rho GEFs in metabolism and metabolic diseases. The study of Rho GTPases in glucose homeostasis has been limited by the wide-ranging essential cellular functions of these proteins, which mean that long-term downregulation or overexpression are prone to inducing non-physiological effects. Dominant-negative or constitutively active mutants are no longer widely used for similar reasons. Rho GTPases function by protein/protein interaction, and pharmacological inhibition is difficult to achieve with high specificity and efficacy. Some bacterial toxins are highly efficient inhibitors, but with considerable cytotoxic effects. Increased use of cell type-specific inducible deletion of the endogenous GTPase, as well as re-expression of mutants to physiological levels, may advance our understanding of the roles Rho GTPases play in metabolic processes, both in isolated cells and in animal models of metabolic disease. Although great steps have been taken to decipher the roles of Rho-GEFs in metabolism, these roles have been restricted to the relatively few examples discussed. Research should focus on other processes such as hormone secretion from the intestine, and the counter-regulatory responses to low glucose levels by glucagon. Global phosphoproteomic analysis of myoblasts differentiated from type 2 diabetic patient iPS cells, has recently revealed cell autonomous insulin resistance and dysregulation of a vast signaling network that goes beyond canonical insulin signaling [193]. There was reduced phosphorylation of proteins involved in Rho GTPase regulation, including the GEFs ARHGEF18 and ARHGEF10 and the Rac1 GAP ARHGAP17, whilst there was downregulation of DOCK7 [193]. These are exciting proteins for further research, as they may impact the actin cytoskeleton controlling glucose uptake, or indeed regulate glucose homeostasis through cytoskeleton-independent mechanisms. 

The study of Rho GEFs promises to yield insights into specific signaling inputs into Rho GTPases in glucose homeostasis. As we have described, several Rho GEFs have been implicated in the regulation of glucose-stimulated insulin secretion in β cells (Figure 5), and in the regulation of GLUT4 translocation and glucose uptake in skeletal muscle and adipose tissue in response to various stimuli, amongst other processes. However, most work has been performed in vitro, and there is a distinct lack of in vivo experiments to show the true physiological relevance of both Rho GTPases and Rho GEFs in metabolism. Among the 10 Rho-GEFs that have been identified to date, the roles of P-Rex2, Vav2, Vav3 and PDZ-RhoGEF in glucose homeostasis mechanisms have so far been investigated using mouse models with whole-body deletions, and only Vav2 has been investigated using mouse models of mutants with low or high GEF activity. Hence, there is massive scope for important in vivo studies in the future. In particular, sophisticated mouse models with inducible deletion of GEFs in specific organs or cell types, or inducible expression of GEF mutants would be desirable. In addition, the knowledge gap regarding potential roles of Rho GEFs in glucoregulatory responses of the liver should be the focus of research groups going forward. It is yet to be determined how translatable the in vivo evidence from mouse models is to the human system, as there are few human studies supporting the mouse work, one of these rare examples being the downregulation of P-Rex2 in adipose tissue of insulin resistant patients [129]. 

Whether Rho GEFs can be pharmacologically targeted in metabolic disease is only just beginning to be explored. It seems sensible to target the Rho GEF over the Rho GTPase, to regulate with higher specificity for each signal. Whereas roles for Rho-GEFs in metabolic disease are only starting to emerge, the widespread involvement of these proteins in the onset, progression and metastasis of cancerous malignancies is well-documented [194,195]. Therefore, there is already great interest in developing inhibitors against these proteins for therapeutic use [196]. Yet, Rho-GEFs are not traditionally considered ‘druggable’ targets, as they function through protein-protein interaction with their target Rho-GTPase, and such interactions involve large surfaces which are difficult to inhibit with high efficacy and specificity [196]. Rho-GEF inhibitors that have been developed as laboratory tools exemplify this point, as they usually have IC_50_s in the micromolar range and limited selectivity for specific GEFs [197]. Most of these compounds target the surface of the catalytic domain of the GEF that interphases with the Small GTPase. However, more indirect modes of inhibition, such as compounds that block the phosphorylation or epigenetic regulation of GEFs, are also being considered. Pharmaceutical companies have recognized the potential of targeting Rho-GEFs and have begun to develop Rho-GEF inhibitors. For example, Novartis is pursuing a Vav inhibitor for the prevention and treatment of graft rejection (patents EP1617868A1 and WO2004091654). Future in vivo studies, combined with the development and testing of Rho-GEF inhibitors, will be required to determine the potential of targeting Rho-GEFs in type 2 diabetes and other metabolic diseases. This is an exciting area for research, which should seek to determine more in vivo translation as well as potential adapter functions of the Rho-GEFs in metabolism.

## Data Availability

No new data were created or analyzed in this study. Data sharing is not applicable to this article.
